# The Gárdos Channel and Piezo1 Revisited: Comparison between Reticulocytes and Mature Red Blood Cells

**DOI:** 10.3390/ijms25031416

**Published:** 2024-01-24

**Authors:** Polina Petkova-Kirova, Nicoletta Murciano, Giulia Iacono, Julia Jansen, Greta Simionato, Min Qiao, Carmen Van der Zwaan, Maria Giustina Rotordam, Thomas John, Laura Hertz, Arjan J. Hoogendijk, Nadine Becker, Christian Wagner, Marieke Von Lindern, Stephane Egee, Emile Van den Akker, Lars Kaestner

**Affiliations:** 1 Institute of Neurobiology, Bulgarian Academy of Sciences, 1113 Sofia, Bulgaria; kirovaps@yahoo.com; 2 Department of Biochemistry, Saarland University, 66123 Saarbrücken, Germany; 3 Nanion Technologies, 80339 Munich, Germany; nicoletta.murciano@nanion.de (N.M.); giustina.rotordam@nanion.de (M.G.R.); nadine.becker@nanion.de (N.B.); 4 Theoretical Medicine and Biosciences, Campus University Hospital, Saarland University, 66421 Homburg, Germany; julia.jansen26@gmx.de (J.J.); min.qiao@uni-saarland.de (M.Q.); laurahertz@gmx.de (L.H.); 5 Department of Hematopoiesis, Sanquin Research, 1066 CX Amsterdam, The Netherlands; g.iacono@sanquin.nl (G.I.); c.vanderzwaan@sanquin.nl (C.V.d.Z.); a.hoogendijk@sanquin.nl (A.J.H.); m.vonlindern@sanquin.nl (M.V.L.); e.vandenakker@sanquin.nl (E.V.d.A.); 6 Landsteiner Laboratory, Amsterdam UMC, University of Amsterdam, 1007 MB Amsterdam, The Netherlands; 7 Department of Experimental Physics, Saarland University, 66123 Saarbrücken, Germanythomas.john@uni-saarland.de (T.J.); christian.wagner@uni-saarland.de (C.W.); 8 Department of Experimental Surgery, Campus University Hospital, Saarland University, 66421 Homburg, Germany; 9 Physics and Materials Science Research Unit, University of Luxembourg, L-1511 Luxembourg, Luxembourg; 10 Biological Station Roscoff, Sorbonne University, CNRS, UMR8227 LBI2M, F-29680 Roscoff, France; egee@sb-roscoff.fr; 11 Laboratory of Excellence GR-Ex, F-75015 Paris, France

**Keywords:** erythrocytes, reticulocytes, patch clamp, membrane potential, NS309, Yoda1, TRAM34, calcium

## Abstract

The Gárdos channel (KCNN4) and Piezo1 are the best-known ion channels in the red blood cell (RBC) membrane. Nevertheless, the quantitative electrophysiological behavior of RBCs and its heterogeneity are still not completely understood. Here, we use state-of-the-art biochemical methods to probe for the abundance of the channels in RBCs. Furthermore, we utilize automated patch clamp, based on planar chips, to compare the activity of the two channels in reticulocytes and mature RBCs. In addition to this characterization, we performed membrane potential measurements to demonstrate the effect of channel activity and interplay on the RBC properties. Both the Gárdos channel and Piezo1, albeit their average copy number of activatable channels per cell is in the single-digit range, can be detected through transcriptome analysis of reticulocytes. Proteomics analysis of reticulocytes and mature RBCs could only detect Piezo1 but not the Gárdos channel. Furthermore, they can be reliably measured in the whole-cell configuration of the patch clamp method. While for the Gárdos channel, the activity in terms of ion currents is higher in reticulocytes compared to mature RBCs, for Piezo1, the tendency is the opposite. While the interplay between Piezo1 and Gárdos channel cannot be followed using the patch clamp measurements, it could be proved based on membrane potential measurements in populations of intact RBCs. We discuss the Gárdos channel and Piezo1 abundance, interdependencies and interactions in the context of their proposed physiological and pathophysiological functions, which are the passing of small constrictions, e.g., in the spleen, and their active participation in blood clot formation and thrombosis.

## 1. Introduction

For a long time, it has been unclear if ion channels in red blood cells (RBCs) have a physiological function or are just relics [1,2]. This may be attributed to the low copy number of ion channels in the RBC membrane, along with functional ion channel measurements without knowing the ion channels’ molecular identity [3,4] and the abundance of channels at or even below the detection limit of classical biochemical methods [5,6]. Further, a persistent rumor for channels simply being relics from cellular development remains, despite accumulating evidence about the contribution of ion channels to RBC flow properties, especially passing constrictions and their active contribution to thrombus and clot formation [7,8].

Here, we present a further investigation of the Gárdos channel and Piezo1 because these are the best-known channels in RBCs in the sense that they are increasingly described to be involved in biophysical–biochemical coupled processes in RBC physiology and pathophysiology [9,10,11]. This, however, does not mean that we already know all their regulation and interplay properties in RBCs, which can be distinctly different from the ones in other cell types [12].

The Gárdos channel was the first channel discovered in (human) RBCs, taking advantage of the patch clamp method [13,14]. These measurements were based on previous reports of Ca^2+^-induced K^+^ loss in RBC suspensions [15,16]. Numerous reports of single-channel recordings followed the initial description of the channel [17,18,19,20,21,22,23,24]. Finally, the Gárdos channel was identified at the molecular level as KCNN4 (hSK4, K_Ca_3.1, IK1) [25]. However, in the RBC community, the KCNN4 channel is still referred to as the Gárdos channel [4,26]. In recent years, with the advent of next-generation sequencing, mutations in the Gárdos channel were discovered and associated with Hereditary Xerocytosis (HX) and, more recently, more specific Gárdos Channelopathy, e.g., [11,27,28,29,30,31,32,33]. The copy number of Gárdos channels per cell is believed to be rather low (25% of the cells contain 11–55 copies and 75% of the cells 1–5 channels according to Grygorczyk et al. [18] and, on average, 2.6 channels per cell according to Wolff et al. [34]). On the other hand, the Gárdos channel provides a significant and measurable effect (Ca^2+^-induced K^+^ loss). Regularly, the K^+^ loss is accompanied by Cl^−^ loss through Cl^−^ channels for the sake of electroneutrality. As a consequence, Gárdos channel activity is mostly accompanied by water loss and, hence, RBC dehydration. However, this process depends critically on the mode of the initial Ca^2+^ entry. If the Ca^2+^ entry is caused by the activity of a non-selective cation channel, the Ca^2+^ entry is accompanied by a Na^+^ entry. This Na^+^ uptake may counteract or even overcompensate for the K^+^-induced dehydration.

The natural history of Piezo1 in RBCs was distinctly different compared to the Gárdos channel. Piezo1 and Piezo2 were identified as the molecular components of mechanosensitive ion channels in general [35,36]. Next, the identification of mutations of Piezo1 in patients suffering from HX proved the abundance of Piezo1 in RBCs [37,38,39], because the HX patients presented a hematological phenotype with altered membrane permeability and Ca^2+^ signaling. There was further functional/pharmacological (mainly based on the application of the agonist Yoda1 [40]) and molecular evidence for the abundance of Piezo1 in RBCs [41,42,43,44,45]. Interestingly (similar to the Gárdos channel), it became evident that Piezo1 was recorded in RBCs even before its molecular identity was discovered [46,47].

Furthermore, transgenic animal models and microfluidic assays increased our understanding of the contribution of the interplay between the Gárdos channel and the mechanosensitive channel Piezo1 in the volume adaptation required for RBCs to pass constrictions within the circulation [48,49,50].

Within the circulation, healthy RBCs show a very high morphological similarity [51], whereas functional aspects can appear very heterogeneous [52,53]. However, in terms of populations, we only discriminate between reticulocytes (very young RBCs defined either by the presence of intracellular mRNA or the abundance of the transferrin receptor on the membrane) and mature RBCs (erythrocytes). A certain exception is the so-called neocytes, which are less well-defined and seem, in terms of classification, to be of limited physiological relevance [54,55].

In this context, we aimed to investigate the function and abundance of both the Gárdos channel and Piezo1, in mature RBCs in comparison to reticulocytes and discuss their interaction and physiological function.

## 2. Results

### 2.1. Biochemical Analysis

**Figure 1:**
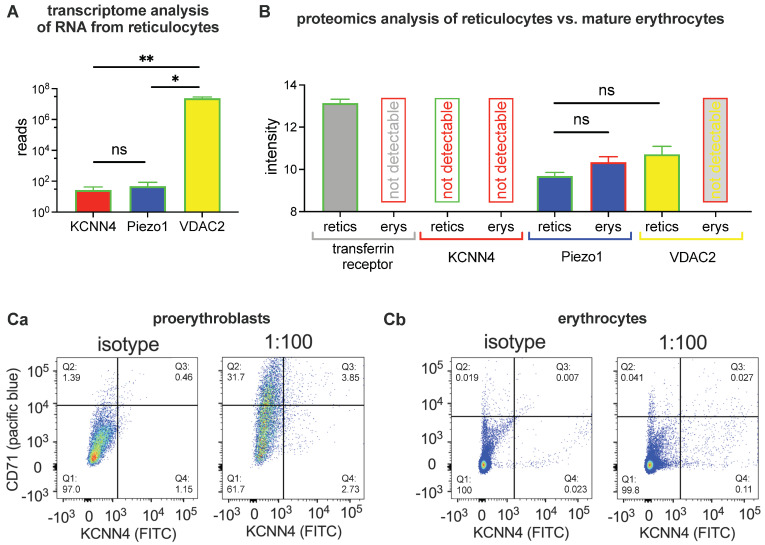
**Transcriptome and protein analysis.** Panel (**A**) shows the transcriptomic analysis for the Gárdos channel (KCNN4), Piezo1 and VDAC2 based on RNA isolated from circulating reticulocytes. The columns present the mean values from 4 donors with the error bars being the standard error of mean (SEM). Gárdos channel (KCNN4) is slightly lower in the reads compared to Piezo1 but the difference is not significant whereas both of them are significantly smaller than VDAC2. Comparisons of the means were tested depicting a *p* > 0.05 for not significant (ns), * for *p* < 0.05 and ** for *p* < 0.01. Panel (**B**) shows the result of the proteomic analysis performed on highly enriched reticulocyte (retics) and reticulocyte-depleted mature RBCs (erys) lysates from 4 different donors with the error bars being SEM. In reticulocytes, the transferrin receptor, Piezo1 and VDAC2 could be detected, whereas the Gárdos channel was below the detection limit. In mature RBCs only Piezo1 could be detected. Comparisons of the means were tested depicting a *p* > 0.05 for not significant (ns). Panel (**Ca**) shows a dot plot of Gárdos channel antibody (KCNN4, FITC) vs. transferrin receptor (CD71, pacific blue) for proerythroblasts presenting a ‘positive control’ for the isotype vs. antibody stains. Panel (**Cb**) shows the same measurements (but different gain settings) for RBCs presenting the reticulocytes in sectors Q2 and Q3.

We performed a transcriptome analysis based on RNA isolated from reticulocytes. Figure 1A shows the outcome for the Gárdos channel and Piezo1 compared to VDAC2 [56] as a positive control. Please note the logarithmic scale in Figure 1A.

We also performed a proteomic analysis of RBCs. Figure 1B shows the mass spectrometry values in arbitrary intensity values for reticulocytes and mature RBCs. The data present the average of four donors. Reticulocytes were enriched based on a CD71 (transferrin receptor) microbead preparation, leading to 72 ± 5% of the enriched cells being classified as reticulocytes by the reticulocyte marker thiazol-orange in flow cytometry. For the mature RBC population, 99.4 ± 0.3% could be classified as thiazol-orange negative. Mass spectrometry revealed the detection of 1461 proteins for the enriched reticulocytes and 845 proteins for the reticulocyte-depleted mature RBCs. In Figure 1B, we show an additional quality control of the transferrin receptor, which is highly abundant in reticulocytes but not detectable in mature RBCs. The Gárdos channel (KCNN4) is detectable in neither reticulocytes nor in mature RBCs. Piezo1 can be reliably detected in both preparations of reticulocytes and mature RBCs. Surprisingly, the detection intensity is higher in mature RBCs compared to reticulocytes, although this difference is not statistically significant (*p* = 0.2). In analogy to the transcriptomic data, VDAC2 is plotted in Figure 1B. Although the RNA reads of VDAC2 in reticulocytes are several orders of magnitude higher than for Piezo1 (Figure 1A), the proteomic intensity of VDAC2 is of comparable intensity as for Piezo1 (*p* = 0.2). In mature RBCs, VDAC2 was below the detection limit. Of note, other preparation methods of RBCs, e.g., preparations of RBC ghosts likely show altered results in the protein detection. This dependence of the detection results on the cell preparation mode is a challenge in the proteomic analysis, and it highlights a general challenge in RBC-related ion channel research [3].

To further illustrate this aspect, in the next step, we assessed cells with fluorescently labelled antibodies against the Gárdos channel and analyzed them in a flow cytometer. Figure 1C shows dot plots of staining for the transferrin receptor (CD71, pacific blue) vs. the Gárdos channel (KCNN4, FITC) for proerythroblasts (Figure 1(Ca)) and peripheral RBCs (Figure 1(Cb)). The comparison with the isotype reveals that the number of KCNN4 positive cells (Q3 + Q4)_antibody_ − (Q3 + Q4)_isotype_ is with approximately 5% for proerythroblasts, and 1% for erythrocytes rather low. In summary, the abundance of the Gárdos channel is paltry, and the method does not allow for a reasonable quantification.

### 2.2. Patch Clamp Analysis

The patch clamp technique is the most direct approach to decypher ion channel functions as it directly measures the electrical current carried by ions passing the pore of the channel of interest. To ensure specificity in the measured current (channel) (or it could be said also to differentiate the current of interest), internal and external solutions of specific composition, particular voltage protocols and additional chemical or physical stimulation were applied.

**Figure 2:**
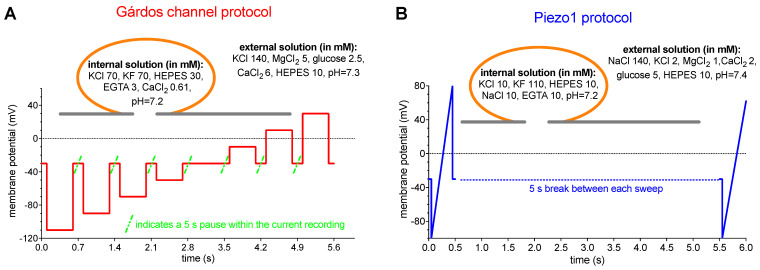
**Patch clamp measurement voltage protocols—a conceptual approach.** Panel (**A**) shows the “voltage-step protocol” as applied for the Gárdos channel measurements. In panel (**B**) the “ramp protocol” as used for Piezo1 recordings is shown. The inserts in both panels depict the contents of the internal and external solutions used for the measurements. The orange line symbolizes the cell and the grey line the planar chip.

We based our measurements on previously reported protocols used for Piezo1 and the Gárdos channel [30,45], with slight modifications. The nature of the Gárdos channel and Piezo1 is vastly different and, therefore, necessitated different voltage protocols, which are outlined in Figure 2.

A “voltage-step” protocol was used to measure the Gárdos channel and a “ramp” protocol to measure Piezo1. Ramps have the advantage of generating current–voltage relations directly and very rapidly and are especially suitable for studying rapidly activating currents. Step protocols measure the steady-state current at a given voltage and, depending on the duration of the step, allow to analyze the kinetics of the current at each voltage and phenomena, such as inactivation and desensitization. Due to the small number and single channel conductance of the Gárdos channels in RBCs [18,34], additional to its evaluation by considering the mean current at a certain voltage, an equally accurate assessment of the channel is given by analysis of the kinetics of the current [30]. A macroscopic whole-cell current, being the result of the summation of many smaller unit currents flowing through single ion channels, exhibits fluctuations about its mean level, and those fluctuations are especially obvious when the Gárdos channel is activated by NS309. Thus, a “voltage-step” protocol was considered for recording Gárdos channel currents (Figure 2A), in contrast to Piezo1, which is a fast inactivating channel (although less fast when activated with Yoda1) [40,57], a “ramp protocol” was the more appropriate choice (Figure 2B).

**Figure 3:**
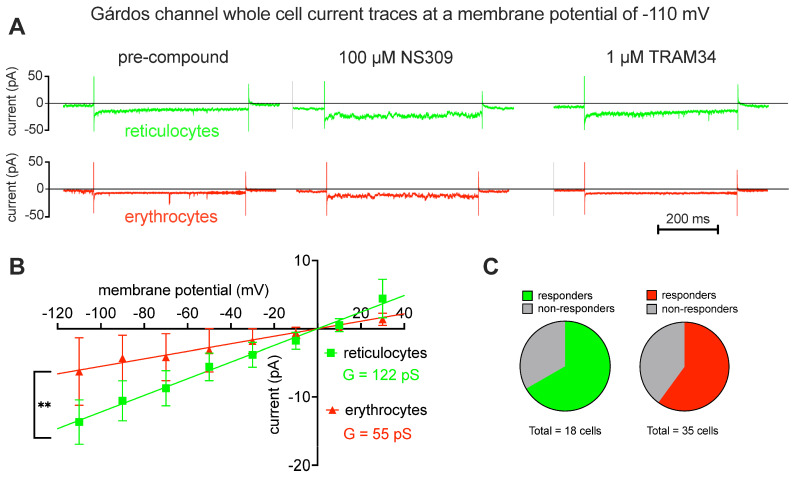
**Gárdos channel measurements.** Panel (**A**) shows representative raw current traces. The first 100 ms of each trace, the voltage is clamped to −30 mV, followed by half a second clamped to −110 mV and then another 100 ms at −30 mV (compare Figure 2A). Green traces refer to reticulocytes and red traces to mature erythrocytes. The left traces are recordings before NS309 application, the middle traces depict channel activity after stimulation with 100 µM NS309, a specific Gárdos channel activator and finally the right traces show the response after application of the Gárdos channel blocker TRAM34 (1 µM). Panel (**B**) provides the current-voltage diagram for reticulocytes (*n* = 13 from 3 donors) and mature erythrocytes (*n* = 10 from 3 donors). Plotted are values of the difference in the mean current with and without NS309 as a read-out of the Gárdos current with error bars representing the standard error of the mean (SEM). Although the Gárdos channel is an inward rectifying channel, in the probed voltage range between −110 mV and 30 mV a linear fit well represents the whole-cell conductance which is 122 pS and 55 pS for reticulocytes and erythrocytes, respectively. The test of significant differences refers to the difference in the slope (conductance). ** indicates a *p*-value below 0.01. Panel (**C**) depicts pie charts indicating the percentage of responding and non-responding cells for reticulocytes (green) and mature RBCs (red).

Figure 3 shows Gárdos channel currents elicited by the “voltage-step” protocol, as outlined in Figure 2A, for reticulocytes and mature RBCs. The pharmacological approach involved measuring the background current before applying a compound (Figure 3A, left traces), stimulating the Gárdos channel by application of NS309 (Figure 3A, middle traces) and, as a last step, blocking the Gárdos channel by application of its inhibitor TRAM34 (Figure 3A, right traces). As a Gárdos channel current is considered to be the difference in currents before and after application of NS309, i.e., the current before application of NS309 being subtracted from the current after application of NS309, and resulting current–voltage curves are given in Figure 3B. Although it is known that the Gárdos channel does not have a linear conductance, in the measured voltage range between −110 mV and +30 mV, a linear regression gives a good approximation of the whole-cell conductance of the channel, and the conductance (slopes in the diagram) was significantly different (*p* = 0.01) between reticulocytes (G = 122 pS) and mature RBCs (G = 55 pS). The percentage of responding cells is provided in the pie charts (Figure 3C), revealing a slightly higher percentage of responding cells in the reticulocyte population compared to the one of mature RBCs.

**Figure 4:**
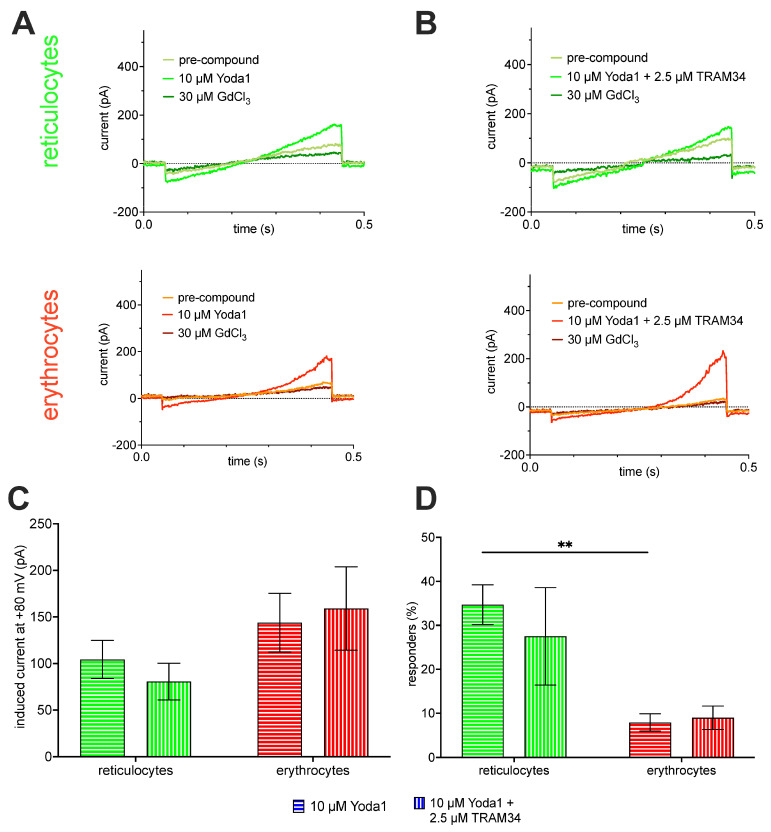
**Piezo1 measurements.** Panel (**A**) shows representative current traces. The first 50 ms of each trace, the voltage is clamped to −30 mV, followed by a 400 ms ramp from −100 mV to 80 mV and then another 50 ms at −30 mV (compare Figure 3B). Green traces refer to cultured reticulocytes and red traces to mature erythrocytes. The recordings show traces before and after stimulation with 10 µM Yoda1, a specific Piezo1 activator and after blockage with 30 µM GdCl_3_. Panel (**B**) shows representative current traces in similarity to panel (**A**), but the stimulation with 10 µM Yoda1 is accompanied by simultaneous inhibition of the Gárdos channel with 2.5 µM TRAM34. Panel (**C**) provides the bar graphs of the Yoda1-induced current for reticulocytes (*n* = 21) from 3 donors and mature erythrocytes (*n* = 20) from 2 donors. Plotted are mean values with error bars representing the standard error of mean (SEM). For explanations of virtually absent contributions of the Gárdos channel response after Piezo1 simulation please refer to the Discussion Section 3.2. The differences in channel activity between reticulocytes and mature RBCs are considered in the Discussion Section 3.3. Panel (**D**) depicts the percentage of responding cells per experiment for reticulocytes (green) from 3 donors and mature RBCs (red) from 2 donors. The test of significance was performed with an ordinary one-way ANOVA with Tukey’s multiple comparisons test. ** refer to a *p*-value below 0.01.

Figure 4 shows Piezo1 measurements applying the “voltage ramp protocol”, as outlined in Figure 2B. The pharmacological concept was to measure the background current before applying a compound (Figure 4A, light green and orange traces for reticulocytes and mature RBCs, respectively). This was followed by stimulation of Piezo1 by application of Yoda1 (Figure 4A, green and red traces for reticulocytes and mature RBCs, respectively) and finally blocking Piezo1 (and putative other non-selective cation channels) by application of the unspecific inhibitor GdCl_3_ (Figure 4A, dark green and dark red traces for reticulocytes and mature RBCs, respectively). Since the activation of Piezo1 results in Ca^2+^-influx, this Ca^2+^ could activate the Gárdos channel; so, the recordings presented in Figure 4A could be the superposition of Piezo1 and Gárdos channel currents. To this end, and to exclude the participation of the Gárdos channel currents, the same experiments were repeated in the presence of the Gárdos channel inhibitor TRAM34 while stimulating the cells with Yoda1 (Figure 4B, same color code as Figure 4A).

Statistical analysis of the current ramps for the voltage of +80 mV is provided in a column diagram (Figure 4C). Although mature RBCs have a smaller surface area compared to reticulocytes, the induced current is bigger for mature RBCs compared to reticulocytes in both experimental conditions (although not significant). The corresponding percentage of responding cells is also given in a column diagram (Figure 4D). The percentage of responding mature RBCs is, on average, below 10% and approximately one-third compared to reticulocytes.

### 2.3. Membrane Potential Measurements

**Figure 5:**
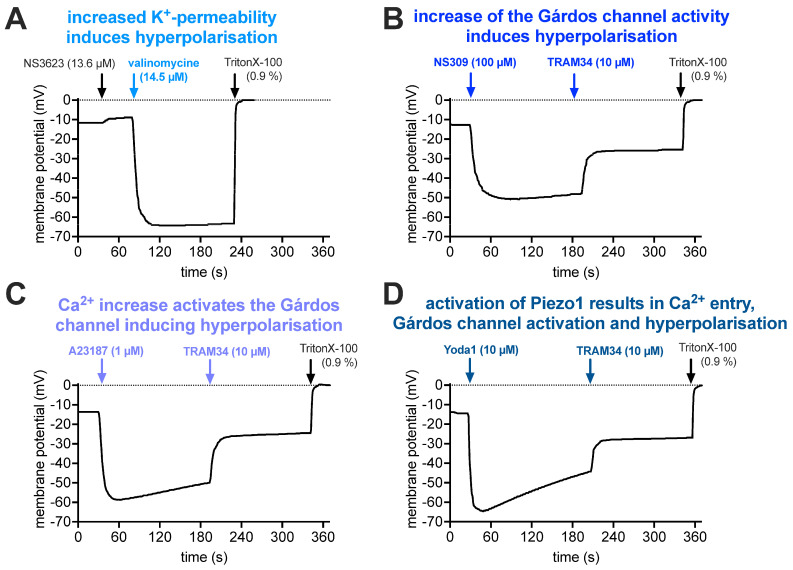
**Effect of Gárdos channel and Piezo1 activation on the membrane potential.** Panel (**A**) represents RBCs, where first the Cl^−^ conductance is blocked with NS3623 and then a K^+^ conductance is induced by the addition of valinomycin. The hyperpolarization can therefore be regarded as fully based on the K^+^ conductance. The experimental solution contained 146 mM NaCl and 9.9 mM KCl. Panel (**B**) shows the hyperpolarization of the cells upon the addition of the Gárdos channel activator NS309. Please note that NS309 only increases the Ca^2+^-sensitivity of the Gárdos channel and hence its open probability, i.e., not all Gárdos channels are open and neither are all RBCs hyperpolarized. The experimental solution was an ordinary Ringer solution (154 mM NaCl and 2 mM KCl). Panel (**C**) shows the hyperpolarization of the RBCs caused by an increase in intracellular Ca^2+^ concentration by the addition of the Ca^2+^ ionophore 4-bromo-A23187 (A23187) inducing the activation of the Gárdos channel. Panel (**D**) shows the hyperpolarization of the cells after activation of Piezo1 by Yoda1, which results in Ca^2+^ entry and hence Gárdos channel activation. All experiments are performed in ordinary Ringer solution, i.e., are nominal Ca^2+^-free, but due to impurities containing an estimated Ca^2+^ concentration of at least 4 µM [58] plus up to 8 µM Ca^2+^ from residual blood plasma (although RBCs were washed). All panels show representative curves of at least duplicate measurements of at least 3 healthy donors.

The electrophysiological characterization of ion channels, as presented in Figure 3 and Figure 4, is, in fact, rather a description of the biophysical properties of the channels, which could be related to function under physiological conditions. To this end, we present drug-induced membrane potential changes (Figure 5), which may successfully indicate channel interactions (in contrast to patch clamp measurements) for intact RBCs.

To interpret the pharmacological stimulations, Figure 5 presents a step-by-step argumentation chain: Figure 5A shows the effect of an increase in the RBC K^+^ permeability. The resting membrane potential of RBCs is approximately −12 mV. The addition of NS3623 blocks the RBC Cl^−^ conductance (which determines the resting potential), which results in a shift in the membrane potential towards 0 mV (equilibrium). Valinomycine acts as a K^+^ pore and, therefore, the membrane potential after the addition of valinomycine could be calculated based on the K^+^ distribution using the Nernst equation:

(1) $$V=-\frac{RT}{zF}\times \ln\frac{{\left[K^{+}\right]}_{in}}{{\left[K^{+}\right]}_{out}}$$

where *R* is the universal gas constant, *T* is the temperature (in Kelvin, here 310 K), *z* is the ionic charge number (*z* = 1 for *K*^+^) and *F* is the Faraday constant, amounting to a constant of 26.7 mV for the first term. [*K^+^*]*_in_* can be taken from the literature [59] to be 102 mM and [*K^+^*]*_out_* from the experimental conditions (cp. legend of Figure 5A) as 9.9 mM. Thus, the membrane potential is calculated as −62.3 mV. This is in good agreement with the −64.2 mV hyperpolarization reached in the measurements. The addition of TritonX-100 is a calibration procedure for a membrane potential of 0 mV. 

In contrast to Figure 5A, which provides a proof of principle for the agreement of theory and experiment, further measurements are designed to show the (more physiological) activity of endogenous channels (mainly Gárdos channel, partly Piezo1). Therefore, in the frame of the experimental requirements, the conditions were set as physiological as possible, i.e., without the addition of the Cl^−^ conductance blocker NS3523.

In Figure 5B, it was tested if cellular hyperpolarization was also reached by activation of the Gárdos channel (Ca^2+^-activated K^+^ channel) by NS309. This is indeed the case; however, the polarization is a bit less pronounced for three reasons: (i) the K^+^ concentration in the external solution is slightly different than in the experiments presented in Figure 5A (compare figure legend); (ii) to assess the physiological effect of the Gárdos channel activation, no additional NS3623 to block the Cl^−^-conductance was applied; and (iii) we measured the average response of all cells, and possibly not all RBCs responded to the NS309 stimulation. Furthermore, inhibiting the Gárdos channel with TRAM34 resulted in a significant depolarization, proving the involvement of the Gárdos channel in the membrane potential jump. For TritonX-100, please refer to Figure 5A. In Figure 5C, we show that a similar Gárdos channel-mediated hyperpolarization can be induced by increasing the intracellular Ca^2+^ concentration, with an application of the Ca^2+^ ionophore A23187, i.e., Ca^2+^ enters the cell and acts as the natural agonist of the Gárdos channel. Again, depolarization upon the addition of TRAM34 proves the contribution of the Gárdos channel. Finally, in Figure 5D, we activate Piezo1, which allows Ca^2+^ entry and, thus, the activation of the Gárdos channel, demonstrating the interplay between Piezo1 and the Gárdos channel. Once more, the addition of TRAM34 induces depolarization.

While in Figure 5A,B, the level of hyperpolarization after its initial induction is stable (constant over time), in Figure 5C,D, it declines over time (more pronounced in Figure 5D). Figure 5C,D rely on Ca^2+^ entry into the RBCs. The Ca^2+^ handling of RBCs is, in general, a complex process [60], and even after stimulation with the ionophore A23187, the Ca^2+^ concentration does not stay constant [61]. In addition, we have to consider that the external Ca^2+^ concentration is very low (well below 15 µM, compare legend of Figure 5). Therefore, we need to consider that a number of RBCs manage to reduce their intracellular Ca^2+^-content below the opening threshold of the Gárdos channel. Since we measured the average membrane potential of the RBCs in suspension, slow depolarization becomes detectable. For the activation of Piezo1, the Ca^2+^ entry is more transient compared to the ionophore A23187 and, therefore, less Ca^2+^ enters the RBC, consecutive Ca^2+^ removal is faster and, hence, more cells depolarize. This means that the detected depolarization (Figure 5D) is faster and more pronounced than in Figure 5C.

Furthermore, after application of TRAM34 and the blockage of the Gárdos channel, the membrane potential does not return to the initial resting membrane potential (Figure 5B–D). The value of repolarization achieved is strictly dependent on the (new) equilibrium for Cl^−^ (and, to a lesser extent, Na^+^). The permeabilities for these two ions will determine the new membrane potential. If we consider, in the three panels of Figure 5B–D, a final membrane potential, post TRAM34, of −27 mV and assuming that the conductance of the Gárdos channel (P_K^+^_) is zero, the Na^+^ conductance (P_Na^+^_) is negligible compared with Cl^−^ conductance (P_Cl^−^_), and the Goldman–Hodgkin–Katz equation (Equation (2), see below) for these three ions is simplified to the Nernst equation (Equation (1), see above) for anions. So, based on an extracellular concentration of 156 mM for Cl^−^, a membrane potential change to −27 mV would represent a decrease in the intracellular anion concentration from around 100 mM to 55 mM. Such a decrease is entirely compatible with the anion flux associated with the activation of the Gárdos channel for electroneutrality reasons.

## 3. Discussion

### 3.1. The Copy Number of Ion Channels in Red Blood Cells—Context and Consequences

For the most prominent ion channels in the RBC membrane, the Gárdos channel and Piezo1, we could show that they are still at or, for some approaches, below the detection limit using state-of-the-art biochemical detection methods (transcriptomics, proteomics and antibody-based flow cytometry). The principal proof is in line with patient investigations, showing an explicit hematological phenotype when carrying pathological variants of the Gárdos channel or Piezo1 [11,27,30,31,32,33,37,38,39,45]. The same holds true for transgenic approaches in animal models [42,48,50,62,63]. Of note, there are other ion channels in RBCs with strong functional evidence for their existence, but controversial biochemical detection and electrophysiological measurements are impossible or difficult to realize such as for Ca_V_2.1 [11,64,65,66] or TRPC6 [6,66,67,68].

In all of the examples above, it is the low copy number in the ion channel in the RBC membrane that is the major obstruction, mainly because RBCs lack protein translation [69]. Thus, there is a necessity to discriminate between the physical presence of a protein, which may still be recognized by an antibody, while the function of the protein might be lost at some point in the, on average, 120-day lifetime of an RBC in the circulation [54], or vice versa, when proteins might not be physically detected while functional evidence for their presence is at hand, like in our study, showing below-limit mass spectrometry values for the Gárdos channel.

Considering all these circumstances, it is not surprising that most of the patch- clamp-based reports on RBCs represent cherry-picking results, hiding the success rate and/or yield of measured channels, with very few exceptions, e.g., [18,70]. Particularly, for the Gárdos channel, the majority of published recordings are performed as “inside-out patches” (single channel recordings) and only very few as “whole-cell recordings”, e.g., [30,71], based mostly on patients suffering from hereditary anemias with an, on average, younger RBC population. In this respect, we consider the data presented here as a “round up” of previous reports. Based on the patch clamp measurements presented in this report, we estimated the channel numbers for the Gárdos channel based on the whole-cell conductance (122 pS for reticulocytes and 55 pS for mature RBCs; Figure 3B) and a single channel conductance of 18 pS [13,14], being 6.8 and 3.1 in reticulocytes and mature RBCs, respectively, for cells classified as responders. Considering the proportion of non-responders (33.3% for reticulocytes and 40% for mature RBCs; Figure 3C), the average number of NS309-activatable number of Gárdos channels per cell drops to 4.5 and 1.8 in reticulocytes and mature RBCs, respectively. This is in rough agreement with previous reports [18,34], as outlined in the Introduction. For Piezo1, we have statistical data only for particular voltages such as 80 mV (Figure 4C). Based on a Piezo1 single channel conductance of 29 pS [72], the current of a single channel at 80 mV corresponds to 2.3 pA. This, in turn, results in a Yoda1-activatable number of fully activated channels of 35 and approximately 65 for reticulocytes and mature RBCs, respectively (for a discussion of why mature RBCs show higher activity, see below). Considering the proportion of non-responders (72.5% for reticulocytes and 81% for mature RBCs; Figure 4D), the average of the Yoda1 (in the presence of TRAM34) activatable number of Piezo1 channels per cell drops to 9.5 and 5.8 in reticulocytes and mature RBCs, respectively. Thus, the number of activatable Piezo1 channels is considerably higher than that of activatable Gárdos channels but still within the same order of magnitude. Please note, the number of activatable channels measured using patch clamp may not represent the physical number of channel copies. We believe the main cause for the high number of non-responding RBCs is the loss of the composition of the intracellular cytoplasm in combination with the low copy number of channels and the stochastic opening behavior of the channels.

The estimated channel number from the patch clamp recordings is in agreement with the proteomic approach (Figure 1B), which compares reticulocytes and mature RBCs as well as the Gárdos channel and Piezo1. Patch clamp indicates a smaller copy number for the Gárdos channel compared to Piezo1; for the proteomics approach, Piezo1 is detectable, whereas the Gárdos channel is below the detection limit for both reticulocytes and mature RBCs. Even more interesting is the comparison of Piezo1 between reticulocytes and mature RBCs, which is discussed in further detail in Section 3.3, below.

**Figure A1:**
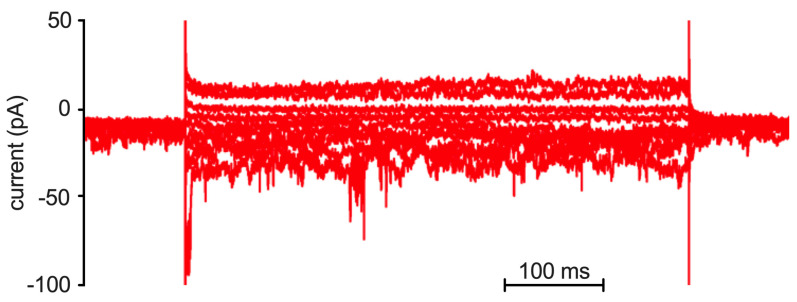
**Example of patch clamp recording of a red blood cell.** Overlay of currents at various potentials (cp. Figure 2A) while stimulated with the Gárdos channel activator NS309. Although this is a whole-cell recording, single channel openings are visible in the flickering appearance of the current traces.

The activity of the Gárdos channel clearly results in cellular hyperpolarization (Figure 5B–D), i.e., “switching on” the Gárdos channel hyperpolarizes the RBCs and “switching it off” depolarizes the RBC again. While Ca^2+^ entry triggers a synchronized opening of the Gárdos channel and, hence, hyperpolarization (Figure 5C,D), a physiological process for a synchronized simultaneous closing of the Gárdos channel is not known. Interestingly, the low copy number of activatable channels enables membrane potential jumps, hyperpolarization and also depolarization [73]. The reason is the combination of the low copy number of the Gárdos channels with the stochastic nature of the channel openings. Figure A1 shows patch clamp recordings of currents that cause such membrane potential jumps, i.e., the recordings although “whole-cell” in the experimental operation, show single channel (or low channel number) activity. In analogy to action potentials but considering the non-excitable nature of RBCs, we call this phenomenon ‘pseudo action potentials’ (PAPs). In turn, PAPs enable the activation of voltage-gated channels [11,74].

### 3.2. Ion Channel Interactions in Red Blood Cells

**Figure 6:**
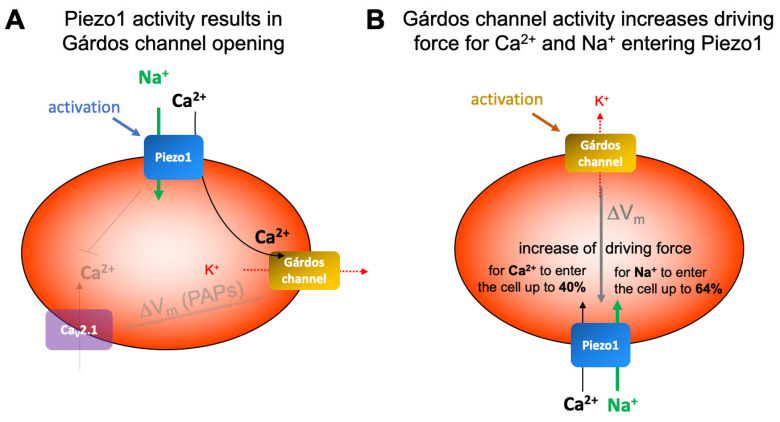
**Schematic presentation of the interplay of Gárdos channel and Piezo1.** Panel (**A**) shows the impact of Piezo1 on the Gárdos channel and in opaque the effect of the Gárdos channel (and to some extent the Piezo1) on the voltage-activated calcium channel Ca_V_2.1 [11,64,65,66]. Panel (**B**) indicates the putative effect of Gárdos channel openings on Piezo1. For further details see main text.

As mentioned above, the activity of the Gárdos channel may drive voltage-activated channels, which, in turn, can be suppressed by Piezo1 activity [11,74] (compare opaque elements in Figure 6A). However, next, we will focus the discussion on the dualism between the Gárdos channel and Piezo1 (Figure 6). 

The hypothesis that Piezo1 activity, which leads to a Ca^2+^ entry, is followed by Gárdos channel activation (Figure 6A) is, in the meantime, an established concept [9,42,48,49,50,75]. Can this also be recognized in patch clamp measurements? In inside-out patches, where only a membrane patch covers the pipette tip, the volume in the bath is so much higher than in the cell, and without diffusion barriers, the dilution of the Ca^2+^ entering through Piezo1 is immediate; hence, no activation of the Gárdos channel is possible. Regarding whole-cell measurements, although the inner RBC volume is connected to—again—the very large volume of the patch pipette (or the equivalent in the planar chip), activation of the Gárdos channel must be considered, and, therefore, all our Piezo1 measurements were performed in the absence and presence of the Gárdos channel inhibitor TRAM34 (Figure 4). While for the reticulocytes, we saw a small insignificant current decrease in the presence of TRAM34, which might be caused by hindering the immediate Ca^2+^ dilution by diffusion barriers (internal structures, e.g., mitochondria), such a decrease was absent in the organelle-free mature RBCs (Figure 4C). However, the lack of Gárdos channel activation following Piezo1 activation in patch clamp measurements was shown before [45] and can be attributed as a patch clamp measurement limitation. In contrast, the MBE measurements in intact cells clearly demonstrate the Gárdos channel opening after Piezo1 activation (Figure 5D). As a side note, Figure 5C,D nicely demonstrate that in the nominal absence of Ca^2+^, the Ca^2+^ “contamination” of solutions, even when using analytical-grade chemicals, we estimate that at least 4 µM plus up to 8 µM from residual blood plasma is sufficient to activate the Gárdos channel [58].

Vice versa, we noticed an increase in current after TRAM34 application, which was also previously reported [45]. Although we do not have a comprehensive explanation for this effect, here, we point to another problem, which arises from the small copy number in the ion channels in RBCs, that is, the abundance of pharmacological side effects. Most drugs cause such side effects, and if the number of channels is very small, the side effects could (in contrast to overexpressing systems, where drugs are usually tested) overwhelm the intended effect of a particular drug, as shown for TRPC6 inhibitors on RBCs [66].

In addition to the effect of Piezo1 on the Gárdos channel (Figure 5D and Figure 6A), there is also an influence of the Gárdos channel on Piezo1, which originates from the hyperpolarization caused by the Gárdos channel (Figure 4C and Figure 5B) and which is visualized in Figure 6B. The electrochemical gradient is the driving force for a particular ion to cross the membrane. It is the sum of the chemical potential, caused by the unequal distribution of the ion on both sides of the membrane, which can be calculated by the Nernst equation (see Equation (1), above) and the electrical driving force, which is caused by the distribution of all ions and their membrane permeability. The electrical driving force was measured in the experiments shown in Figure 5 and can be calculated by the Goldman–Hodgkin–Katz equation (Equation (2), see below). The potentials acting on particular ions are summarized in Table 1 and given for the resting membrane potential (approximately −12 mV) and for the membrane potential after Gárdos channel activation (hyperpolarization, approximately −70 mV)—compare with Figure 5.

However, are the potentials given in Table 1 applicable for ion transport through Piezo1? The situation is a bit more complex, because in the moment that Piezo1 is open, the membrane potential is also influenced by the permeability changes caused by Piezo1. Therefore, the electrochemical potentials given in Table 1 denote the boundaries within the membrane that the potential may adjust. The actual membrane potential is defined by the Goldman–Hodgkin–Katz equation:

(2) $$V_{m}=\frac{RT}{F}\times \ln\frac{\sum_{i}^{n}P_{M_{i}^{+}\;}{\left[M_{i}^{+}\right]}_{out}+\sum_{j}^{m}P_{A_{j}^{-}}{\left[A_{j}^{-}\right]}_{in}}{\sum_{i}^{n}P_{M_{i}^{+}\;}{\left[M_{i}^{+}\right]}_{in}+\sum_{j}^{m}P_{A_{j}^{-}}{\left[A_{j}^{-}\right]}_{out}}$$

where *M*^+^ is monovalent cations and *A*^−^ is monovalent anions, with *P* being the permeability of the indexed ion. For illustrative purposes, Equation (2), just considering Na^+^, K^+^ and Cl^−^ ions, would read:

(3) $$V_{m}=\frac{RT}{F}\times \ln\frac{P_{Na}{\left[{Na}^{+}\right]}_{out}+P_{K}{\left[K^{+}\right]}_{out}+P_{Cl}{\left[{Cl}^{-}\right]}_{in}}{P_{Na}{\left[{Na}^{+}\right]}_{in}+P_{K}{\left[K^{+}\right]}_{in}+P_{Cl}{\left[{Cl}^{-}\right]}_{out}}$$

In these equations, the permeability *P* is crucial, i.e., the distinct number of available functional Piezo1 and Gárdos channels determines the cell permeability and, hence, the membrane potential.

To better judge the situation, we include one more aspect in the discussion, which is the opening time (interval) of the channels. As mentioned before, Piezo1 has a transient opening behavior. In intact RBCs, this transient opening is obviously sufficient to have enough Ca^2+^ to enter the cell for an activation of the Gárdos channel. The dominating K^+^-loss leads to volume adaptations in particular cell shrinkage, e.g., to allow RBCs to pass small capillaries or the sinusoidal slits in the spleen following mechanical stress [48,49,50,81]. Although Piezo1 and TRPV2 share a number of properties [82], such as being nonselective cation channels and having the same magnitude of the single channel conductance, the activation of the two channels has different consequences. The activation of TRPV2, e.g., by Δ^9^-tetrahydrocannabinol [83], results in longer opening times, and although Ca^2+^ is expected to enter the RBC and activate the Gárdos channel, due to different driving forces (compare Table 1), the Na^+^ entry trough TRPV2 dominates the K^+^ exit through the Gárdos channel with the result that RBCs swell [84,85]. This shows that (patho)physiological effects can be tuned in opposite directions by a single biophysical property of a channel, such as the transient nature of the opening.

### 3.3. Ion Channel Differences between Reticulocytes and Mature Red Blood Cells

We do see differences in ion channel function between reticulocytes and mature RBCs for both channels, the Gárdos channel and Piezo1, but opposite trends.

For the Gárdos channel, even when considering that reticulocytes have, on average, a 20% bigger surface area than mature RBCs [80], still, the current density is double the size in reticulocytes compared to mature RBCs. In accordance with this, the number of responding cells drops from 67% to 60%. This decrease in activity is somehow expected because of the lack of protein translation and protein “aging” [69]. It is also in line with data in the literature, showing age-dependent changes in RBC composition, metabolism and transport [86,87,88].

Surprisingly, the trend of the current density differences between reticulocytes and mature RBCs for Piezo1 is opposite to the Gárdos channel, i.e., increasing in mature RBCs, albeit the mature RBCs contain 20% less membrane [80]. Interestingly, the proteomic approach mirrors the patch clamp experiments with a lower intensity for Piezo1 in reticulocytes compared to mature RBCs (Figure 1B). Because both of these differences are not statistically significant, one should be careful with an “over interpretation”. However, the fact that both conceptually different investigations point in the same direction is remarkable.

A plausible explanation arises from the 20% loss of membrane when reticulocytes mature, which could lead to a rather relative increase. This would mean that Piezo1 is (in contrast to the transferrin receptor and presumably VDAC2; Figure 1B) excluded from the shed membrane during the reticulocyte maturation process. In consequence, this could be a strong argument for the active conservation of Piezo1 in RBCs and explicitly not being a remnant from earlier erythroblast stages [89]. A further interpretation arises from the fact that Piezo1 activity is modulated by the lipid composition of the membrane the channel is embedded in [90,91,92]. There is severe lipid remodeling associated with the ageing process, all the way from reticulocytes to senescent RBCs [93]. This remodeling process may, at least partly, explain the cellular heterogeneity indicated by the fairly large error bars in Figure 4C. The protein ageing process used as an argument when describing the Gárdos channel would also apply to Piezo1 and is, indeed, reflected in the number of responding cells, which drops from 35 ± 5% (reticulocytes) to 8 ± 2% (mature RBCs) *p* = 0.002.

### 3.4. Physiological Function of Ion Channels in Red Blood Cells

The physiological function of the Gárdos channel and Piezo1 was not investigated in this study. However, one of the reviewers encouraged us to include this discussion. In vivo investigations of single RBC properties are rather sparse and mostly limited to animal models [49,94]. Therefore, this subsection is, to some extent, speculative in nature.

One of the putative physiological functions of the Piezo1–Gárdos channel interplay is a volume adaptation, when RBCs need to pass small constrictions like the sinoidal slits in the spleen, which was proposed by several groups [48,49,50]. Indeed, there are a number of facts that support this concept: (i) mechanical stress, like RBCs experience when passing small constrictions, activate Piezo1 [95,96]; (ii) the large gradient of Ca^2+^ across the RBC membrane in the order of 20,000 (cp. Table 1) activation of Piezo1 in RBCs results in an increase in intracellular Ca^2+^ [60]; (iii) an increase in intracellular Ca^2+^ activates the Gárdos channel [73]; and (iv) activation of the Gárdos channel results in dehydration [97]. However, the latter point needs to be a bit relativized because Piezo1 is a non-selective cation channel, and its activation also leads to Na^+^ entry, which slightly counteracts the dehydration caused by the K^+^ loss through the Gárdos channel as discussed above. However, since Piezo1 has, in contrast to the Gárdos channel, a fast inactivation, there is a net loss in intracellular water (dehydration). On the other hand, theoretical and experimental investigations showed that RBCs are able to mechanically pass constrictions like the sinoidal slits without the help of molecular signaling and volume adaptations [98]. Also, old RBCs that are likely to have fewer functional ion channels are able to pass the spleen. Without doubt, RBCs are sophisticated well-tuned cells that fulfill (in addition to gas transport) numerous functions. Therefore, we believe a small volume reduction by the Piezo1–Gárdos channel interplay speeds up the RBC passage of the spleen. Vice versa, a delayed passage of old RBCs without volume adaptation allows for easier access of the macrophages to the RBCs, which is vital for the cleavage of old RBCs.

The other physiological function of the Piezo1 and Gárdos channels is linked to the active contribution of RBCs to clot formation and the formation of red thrombus [7,8]. The argumentation chain is very similar to the previous one: RBCs, when caught in the clot/thrombus, experience mechanical stress, which results in RBC dehydration and, in turn, supports clot formation. In addition, Ca^2+^ plays a more dominant role: Ca^2+^ entry is also activated by substances released from activated platelets like lysophosphatidic acid [66] and prostaglandin E_2_ [99]. This substantially increased intracellular Ca^2+^ concentration also triggers other processes, like scramblase activation and cleavage of calpain, which are in support of the clot/thrombus formation [60].

## 4. Materials and Methods

### 4.1. Blood Collection

Blood collection was performed following the Declaration of Helsinki and was approved by the ethics committee of “Ärztekammer des Saarlandes”, permit number 51/18. Blood was collected from healthy donors into heparin tubes by venipuncture, washed and resuspended. The rationale for using heparin as an anticoagulant was that the surrounding Ca^2+^ concentration can be roughly maintained. It seems important to maintain this condition as long as possible since the removal of external Ca^2+^ likely influences ion homeostasis [100]. Washing the full blood samples was carried out at 1800× *g* for 6 min prior to the procedures described below.

### 4.2. Transcriptome Analysis

To purify human RBCs, we followed the method originally developed by Beutler et al. [101]. Blood samples were centrifuged at 1000× *g* for 20 min. Plasma was aspirated and mixed with phosphate-buffered saline (PBS) (1:10). RBCs were washed 3 times (1000× *g*, 5 min) and mixed with PBS (1:1). Filter paper (Whatman No. 4 GE Healthcare, Buckinghamshire, UK) was pressed in a 10 mL syringe (Omnifix Solo Lure, Braun, Germany), and a mixture of 180 mg Sigma- and 180 mg Alpha-Cellulose (Sigma-Aldrich, Saint Louis, MO, USA) suspended in 10 mL PBS was added. After the PBS drained, the syringe was primed with 10 mL of the diluted plasma. Thus, 1 mL of RBCs was added and eluted with 10 mL PBS. Filtered RBCs were, again, washed 3 times in PBS. For the following RNA isolation, RBCs were used immediately.

To evaluate the purification of the RBCs, we used the gelatin zymography technique [102]. This method allows for the detection of contaminations with polymorphonuclear neutrophils (PMNs), a type of leucocytes that cannot be eliminated by washing the blood sample. PMNs are the only type of blood cells that express the matrix metalloproteinase 9 (MMP-9), whose catalytic activity against gelatin can be used as a specific marker.

Briefly, 10 μL of diluted RBC samples (PBS, 1:10) was lysed by adding Zymogram Tris-Glycine SDS Sample Buffer (1:1) (Thermo Fisher Scientific, Waltham, MA, USA), followed by protein separation in 10% Gelatin Protein Gels (Thermo Fisher Scientific, USA) using a nonreducing SDS-PAGE. After separation (2 h, 125 V), gels were incubated for 1 h in Zymogram Renaturing Buffer (Thermo Fisher Scientific, Waltham, MA, USA) under continuous shaking and washed three times in Aqua dest. For activation of the catalytic activity of MMP-9, the gels were incubated for up to 40 h in a digestion buffer containing (in mM) 50 Tris-HCl pH 7.6, 150 NaCl and 10 CaCl_2_. Degradation of gelatin in the gel could be visualized after Coomassie blue staining as white spots.

For leucocyte depletion, we used antibody-coupled magnetic beads. To reduce the number of CD45^+^ cells that needed to be eliminated, we first performed the Ficoll-Paque separation of the blood sample. Blood was diluted with PBS and layered on top of the Ficoll-Paque solution (GE Healthcare, Chicago, IL, USA). After centrifugation (800× *g*, 25 min), plasma and a layer of leucocytes were removed, and RBCs were washed three times in isolation buffer (PBS with 0.1% BSA and 2 mM EGTA). Cells were incubated overnight at 4 °C with the following antibodies: IgG rabbit anti human CD45 (GeneTex Inc., Irvine, CA, USA) [1:40] and IgG rabbit anti human CD15 (Biorbyt, Cambridge, UK) [1:50]. Magnetic beads (Dynabeads sheep anti-rabbit, Thermo Fisher Scientific, Waltham, MA, USA) were washed once in isolation buffer using a DynaMag Holder (Thermo Fisher Scientific, Waltham, MA, USA) and then added to the RBCs (1:1) for a further 120 min. To remove the bead-bound cells, the RBC-Bead-Mix was washed twice in PBS again using the DynaMag Holder. For RNA isolation, we used the RiboPure RNA Purification Kit (Thermo Fisher Scientific, Waltham, MA, USA) and 500 μL of human blood samples, prepared as described before. Subsequently, the alpha and beta globin mRNA, which have the highest expression in reticulocytes, was removed from the total RNA preparations by using the GLOBINclear Kit (Thermo Fisher Scientific, Waltham, MA, USA), according to the manufacturer’s protocol. Transcriptome analysis was performed by Expression Analysis Inc. (Durham, NC, USA) using next-generation sequencing.

### 4.3. Proteomic Analysis

Leucodepleted RBCs were prepared by spinning 10 mL of peripheral blood at 1380× *g* for 5 min. The supernatant and the first layer of RBCs were removed, and the pellet was washed 3 times in PBS. The obtained packed RBCs were washed twice in MACS buffer (0.5% HAS, 10% TNC in PBS) and incubated with CD71 microbeads (Miltenyi Biotec, Bergisch Gladbach, Germany) for 20 min at 4 °C. Stained cells were washed once, suspended in MACS buffer and subjected to MACS magnetic selection. The reticulocyte enrichment was measured by staining cells with thiazole orange (Sigma-Aldrich, Saint Louis, MO, USA) and counting based on flow cytometry. Thus, 2.5 million cells derived from CD71+ selection and CD71− selection were washed 5 times in PBS and subjected to mass spectrometry.

Cell pellets were lysed in 1% sodium deoxycholate (SDC, BioWORLD, London, UK), 10 mM Tris-(2-carboxyethyl)fosfine (Thermo-Fisher Scientific, Waltham, MA, USA), 40 mM Chloroacetamide (Sigma-Aldrich, Saint Louis, MO, USA), 100 mM Tris pH 8, (Life Technologies, Carlsbad, CA, USA) and heated for 5 min at 95 °C. After cooling to room temperature, the samples were sonicated in a sonication waterbath (Branson Ultrasonics, Brookfield, CT, USA) for 10 min. Total protein was measured using a Bradford assay (Biorad, Hercules, CA, USA). Then, 10 µg of protein was diluted in 50 mM Tris pH 8 and digested for 18 h at 25 °C with trypsin/LysC (Thermo-Fisher Scientific, USA) in a 1:20 enzyme-to-protein ratio. After adding trifluoroacetic acid (Thermo-Fisher Scientific, Waltham, MA, USA) to a final concentration of 1%, samples were centrifuged for 5 min at 10,000× *g* to pellet the precipitated SDC. Supernatant was transferred to a new vial, and 500 ng of peptides was loaded on Evotip pure, according to manufacturer’s instructions.

Peptides were separated on an Evosep one (Evosep, Odense, Denmark) with the preset 30 samples-per-day method on a 15 cm Evosep Performance Column (EV-1137, 150 µm I.D., 1.5 µm particle size). Acquisition was performed on a timsToF-HT (Bruker Daltonics, Billerica, MA, USA) mass spectrometer operated in DIA-PASEF mode. Ion mobility accumulation and ramp time were set to 100 ms. Further, 16 DIA windows were set per cycle, ranging from 0.7–1.5 1/k_0_ to 421–1594 *m*/*z*, and the size of DIA windows was set based on precursor density. Collision energy was set as a linear function of the ion mobility (0.6 1/k_0_ = 20 eV, 1.60 1/k_0_ = 59 eV). Raw files were processed in DIA-NN 1.8.1.; proteins and peptides were detected by querying the filtered human Swissprot database (release 2021.22.04). Standard settings were used, using a generated library-based spectra search. Maximum number of variable modifications was set to 2. Protein Interference used was “Protein names (from FASTA)” and quantification strategy “Robust LC (high precision)”. Data were analyzed using R 4.3.0/Rstudio (2023.12.0). Detected proteins were filtered for proteotypic and ≥2 unique peptides per protein, and proteins were quantified in 100% of samples in at least one condition.

### 4.4. PBMC Isolation and Culture

Peripheral blood (~25 mL) was collected in Li-heparin tubes (Sarstedt, Nümbrecht, Germany). PBMCs were isolated using Ficoll Histopaque (density = 1.077 g/mL, 20 °C; GE Healthcare, Chicago, IL, USA) following the manufacturer’s protocol. Remaining RBCs in the cell isolate were lysed (lysis buffer = 155 mM NH_4_Cl, 12 mM KHCO_3_, 0.1 mM EDTA; 10 min at room temperature). PBMCs were cultured as previously described [103]. In short, a two-phase culture system was employed for in vitro erythropoiesis: in the expansion phase, PBMCs (day 0 expansion) were cultured in CellQuin medium supplemented with EPO (2 IU/mL; Prospec, Atlanta, GA, USA), dexamethasone (1 µM; Sigma-Aldrich, Saint Louis, MO, USA) and human stem cell factor (hSCF; 100 ng/mL, ITK diagnostics, Uithoorn, The Netherlands). The differentiation phase was started at day 13 expansion by washing the cells once with PBS and reseeding them in CellQuin supplemented with human plasma (5% *v*/*v*; Octapharma GmbH, Langefeld, Germany), EPO (10 IU/mL), heparin (5 IU/mL; MP Biomedicals™, Santa Ana, CA, USA) and additional holotransferrin (final concentration of 1000 µg/mL; Sanquin, Amsterdam, The Netherlands). All cultures were kept in humidified incubators (at 37 °C, air plus 5% carbon dioxide). Cell concentration was regularly determined using a CASY cell counter (CASY Model TCC, OLS OMNI Life Science, Bremen, Germany). In case cells were shipped, they were suspended in MOD6 buffer (Sanquin, The Netherlands).

### 4.5. Flow Cytometry

Cultured cells or isolated RBCs were washed with PBS (5 min, 600× *g*), stained with primary antibody or reagents (20 min, room temperature), washed and resuspended in flow cytometry buffer (5 min, 600× *g*) and measured in a FACS Canto™II flow cytometer (BD Biosciences, San Jose, CA, USA). Antibodies used were CD71-PB (1:100 dilution; Miltenyi Biotec, Germany) and KCa3.1 (SK4) (1:100 dilution; Abgent, San Diego, CA, USA). Gating was performed against specific isotype controls: anti-mouse isotype control IgG1k-pacific blue (1:200; Biolegend, San Diego, CA, USA) and anti-mouse isotype control IgG1-PE, (1:200; R&D Systems, Minneapolis, MN, USA). The obtained data were analyzed with Flowjo™ (BD Biosciences, San Jose, CA, USA).

### 4.6. Patch Clamp Measurements

Patch clamp measurements were performed with automated systems based on planar chips, the Patchliner for the Gárdos channel assay and the SyncroPatch 384 for the Piezo1 measurements (both: Nanion Technologies, Munich, Germany). Recordings were performed at room temperature using planar borosilicate glass patch clamp chips for the respective devices with resistances of 5–8 MΩ (Patchliner) and 9–12 MΩ (SyncroPatch 384). The internal and external solutions used to measure the Gárdos channel were as follows (in mM): KCl 70, KF 70, HEPES 30, EGTA 3, CaCl_2_ 0.61, pH = 7.2 adjusted with KOH (internal) and KCl 140, MgCl_2_ 5, CaCl_2_ 6, D-glucose 2.5, HEPES 10, pH = 7.3 adjusted with KOH (external). The internal and external solutions used to measure Piezo1 were as follows (in mM): KCl 10, KF 110, NaCl 10, EGTA 10 and HEPES 10, pH = 7.2 adjusted with KOH (internal) and NaCl 140, KCl 4, CaCl_2_ 2, MgCl_2_ 1, Glucose 5 and HEPES 10, pH = 7.3 adjusted with KOH (external).

Gigaseal formation was facilitated by the use of a seal-enhancing solution, as recommended by the Patchliner manufacturer and containing (in mM) NaCl 80, KCl 3, MgCl_2_ 10, CaCl_2_ 35, HEPES 10, pH = 7.3 adjusted with NaOH. The seal-enhancing solution was only used to help obtain the very high gigaohmic contact between the cell and the chip and, in the whole-cell configuration, was replaced by the external solution. Whole-cell configuration was achieved by negative pressure suction pulses between −45 mbar and −150 mbar, and its formation was judged by the appearance of sharp capacitive transients. Whole-cell patch clamp recordings of the Gárdos channel were conducted using voltage steps from −110 mV to 30 mV for 500 ms in 20 mV increments at 5 s intervals, the holding potential being set at −30 mV. Gárdos current differences between erythrocytes and reticulocytes were evaluated with the use of NS-309 (100 µM), a specific activator of the channel. Whole-cell currents of Piezo1 were elicited using a voltage ramp protocol (−100 mV to 80 mV, 450 ms, every 5 s, holding potential −30 mV). After recording a stable baseline current in external solution, cells were exposed to 10 μM Yoda1 (Tocris, Bristol, UK) for ~4 min to investigate the activity of Piezo1 channels, followed by application of 30 μM GdCl_3_ (Sigma-Aldrich, Saint Louis, MO, USA), a non-selective inhibitor of Piezo1. Where indicated, the selective Gárdos channel inhibitor TRAM-34 (2.5 μM, Tocris, Bristol, UK) was employed in combination with Yoda1. Only cells with seal resistance > 0.5 GΩ were used for analysis. The compound-induced current was obtained by subtracting the average of the last 3 sweeps obtained from the compound addition period and the average of the last 10 sweeps obtained from the reference addition period at 80 mV. If the mean current amplitude elicited upon compound addition exceeded 3σ of the mean current amplitude at baseline conditions, a cell was considered as a responder.

### 4.7. The Macey–Bennekou–Egée (MBE) Method

The membrane potential measurements of an RBC population were performed according to a method initially described by Macey et al. [104], further developed and applied by Poul Bennekou, e.g., Braunbæk and Bennekou [58], and kept alive by the laboratory of Stéphane Egée [31]. Therefore, we refer to it as the MBE method.

For each experiment, 1 mL of the Ringer solution (154 mM NaCl, 2 mM KCl) was poured into a 2 mL Eppendorf tube, and a magnetic stir bar (cylindrical of 8 mm length and a diameter of 3 mm, VWR, Radnor, PA, USA) was added. Then, the protonophore carbonyl cyanide m-chlorophenyl hydrazone (CCCP, Sigma-Aldrich, Saint Louis, MO, USA) was added to reach a final concentration of 27 µM. The Eppendorf tube was placed in a 36 °C water bath, and a rotating magnet (990 rpm) was used to stir the sample. Then, the calibrated pH meter (SevenCompact S210, Mettler-Toledo, Giessen, Germany) was immersed in the liquid (In Lab Solids pro-ISM, Mettler-Toledo, Giessen, Germany) to allow for continuous measurements. The pH meter was connected to a personal computer, and the values were measured every second and recorded via self-designed software. One minute after the start of the recording, 150 µL of washed RBCs were pipetted into the Ringer’s solution containing CCCP and, after two minutes, drugs according to the particular protocol were added. At the end of each experiment, TritonX-100 (Sigma-Aldrich, Saint Louis, MO, USA) was added to reach a final concentration of 0.9% to lyse the RBCs in order to calibrate the system for a membrane potential of 0 mV.

The stored data were transferred to Excel (Microsoft, Redmond, WA, USA), and the recorded pH values were translated into membrane potential using the following formula:

(4) $${V(mV)=-61.5\times (pH}_{out}-{pH}_{in})\;$$

where *pH_out_* is the measured pH value during the experiment, and *pH_in_* is the last measured calibration pH value at the end of the recording after cell lysis.

Finally, the membrane potentials were plotted against time in Prism 9 software (Graph Pad, San Diego, CA, USA).

## 5. Summary and Conclusions

Although both channels, the Gárdos channel and Piezo1, are more less at the detection limit of biochemical methods (Figure 1), they can be functionally studied using both the patch clamp technique (Figure 2, Figure 3 and Figure 4) and the MBE method (Figure 5). With the patch clamp technique, differences between reticulocytes and mature RBCs could be detected for both channels (Figure 3 and Figure 4). The activation of Piezo1 results in Ca^2+^-mediated opening of the Gárdos channel but, vice versa, Gárdos channel activity provides changes in the driving force for ions passing Piezo1 (Figure 6).

Channel properties are extensively discussed to better understand their physiological function, which is volume adaptation when passing small constrictions, such as small capillaries or sinusoidal slits of the spleen [49,105,106], as well as the signaling that leads to the active participation of RBCs in clot and thrombus formation [7,8,107,108].

After more than 40 years of patch clamp investigations on human RBCs [13], the current paper clarifies the most basic principles of RBC electrophysiology, comparing reticulocytes with mature RBCs, the activity of the Gárdos channel (KCNN4, Figure 3), the activity of Piezo1 (Figure 4) and their interaction (Figure 5 and Figure 6), namely the activation of the Gárdos channel triggered by the Ca^2+^ entry mediated by Piezo1. However, the activity of the Gárdos channel also has a feedback mechanism to Piezo1 through the change in membrane potential.

## Figures and Tables

**Table 1:** Driving forces for particular cations for the measured resting membrane potential (−12 mV) and after opening of the Gárdos channel (−70 mV).

Ion	Ca^2+^	Na^+^	K^+^
extracellular concentration	1.2 mM [76]	140 mM [77]	4 mM [78]
intracellular concentration	60 nM [79]	7.35 mM [80]	102 mM [59]
chemical potential *	−132 mV	−79 mV	86 mV
electrochemical potential at resting membrane potential	−144 mV	−91 mV	74 mV
electrochemical potential when Gárdos channel is open	−202 mV	−149 mV	16 mV

* The chemical potential was calculated using the Nernst equation (Equation (1)). A negative potential in the table means the cations enter the cell and a positive potential means the cations exit the cell.

## Data Availability

All relevant data are included in the article; further inquiries can be directed to the corresponding author. Source data files for the figures are provided in the Supplementary Material file.
